# On the difficulty to think in ratios: a methodological bias in Stevens’ magnitude estimation procedure

**DOI:** 10.3758/s13414-021-02266-5

**Published:** 2021-03-31

**Authors:** Alica Mertens, Ulf K. Mertens, Veronika Lerche

**Affiliations:** Institute of Psychology, Heidelberg University, Hauptstrasse 47-51, D-69117 Heidelberg, Germany

**Keywords:** Psychophysics, Stevens’ power law, Magnitude estimation, Bayesian inference

## Abstract

In the field of new psychophysics, the magnitude estimation procedure is one of the most frequently used methods. It requires participants to assess the intensity of a stimulus in relation to a reference. In three studies, we examined whether difficulties of thinking in ratios influence participants’ intensity perceptions. In Study 1, a standard magnitude estimation procedure was compared to an adapted procedure in which the numerical response dimension was reversed so that smaller (larger) numbers indicated brighter (darker) stimuli. In Study 2, participants first had to indicate whether a stimulus was brighter or darker compared to the reference, and only afterwards they estimated the magnitude of this difference, always using ratings above the reference to indicate their perception. In Study 3, we applied the same procedure as in Study 2 to a different physical dimension (red saturation). Results from Study 1 (*N* = 20) showed that participants in the reversal condition used more (less) extreme ratings for brighter (darker) stimuli compared to the standard condition. Data from the unidirectional method applied in Study 2 (*N* = 34) suggested a linear psychophysical function for brightness perception. Similar results were found for red saturation in Study 3 (*N* = 36) with a less curved power function describing the association between objective red saturation and perceived redness perception. We conclude that the typical power functions that emerge when using a standard magnitude estimation procedure might be biased due to difficulties experienced by participants to think in ratios.

## Introduction

Over the last centuries, an impressive effort has been made to unmask the association between the physical intensity of a stimulus and its perceived intensity. Based on Weber's observations on just noticeable differences (JNDs), Fechner (1860) mathematically formulated that the perceived intensity of a stimulus is proportional to the logarithm of the ratio of the physical intensity and the absolute threshold of the respective stimulus. By determining the increases in stimulus intensity that are necessary to cause JNDs in sensation, Fechner claimed to have developed an indirect measure of perceived stimulus intensity (Warren & Warren, 1963). Many decades later, Stevens (1957) introduced the era of the “new psychophysics,” claiming the possibility to measure sensations directly. Developing techniques such as the magnitude estimation approach, which represented one of the most common methods for measuring physical sensations (McKenna, 1985), Stevens found evidence that the psychophysical function translates into a power law (rather than into a logarithmic function, as assumed by Fechner). The general form of this power law is

$$ \psi (I)=k{I}^a, $$

where *ψ*(*I*) is the perceived intensity of a physical stimulus magnitude *I* and *a* is the exponent that determines the form of the psychophysical function. If *a* < 1, the perceived intensity changes less than the actual intensity (compression), whereas *a* > 1 corresponds to the opposite effect (expansion). A value of *a* = 1 indicates a linear relationship. The parameter *k* represents a proportionality constant.

Parameters of the power law are often estimated by fitting the power law to data from the so-called magnitude estimation procedure. In this procedure, participants rate the perceived stimulus intensity (e.g., brightness) in relation to a reference stimulus. Thus, they are required to think in ratios. For example, the value 10 is arbitrarily assigned to a (constant) reference stimulus.^1^ The participants are then asked to indicate the intensity of test stimuli compared to this reference stimulus. For a test stimulus that is perceived to be ten times as bright as the reference, a value of 100 (i.e., 10 × 10) should be entered. A perceived brightness of only one-tenth of the reference should be indicated by a value of 1 (i.e., 10/10). Regarding the relationship between objective (physical) luminance and subjective (perceived) brightness, generally a limited (concave) growth curve is found with an exponent smaller than 1. Stevens (1966) concluded that in the lower range of the luminance continuum differences are perceived as being more pronounced compared to those in the upper area of the luminance continuum.

Although the magnitude estimation approach seems straightforward in the first place, criticism was raised regarding the adequacy and validity of this kind of measurement (e.g., Augustin, 2008; Prytulak, 1975; Weiss, 1981). For brightness estimation, Freides and Phillips (1966) noticed that the power function worked well when using group data, but different results emerged on the individual level. Moreover, Hood and Finkelstein (1979) found that their data could not be fitted by a power function, and Marks (1974a, 1974b) observed that the power law depends on the range of numbers used by the participants. Another problem – raised by Weiss (1981) – is that the resulting function is not only influenced by the participant’s responses, but also by the way the stimuli have to be estimated (e.g., on a continuum, categorically, etc.). When using other methods, like category scaling, a power function is not obtained in most cases (McKenna, 1985).

In the present work, we examine another principal problem of the estimation approach that – to our knowledge – has not yet been systematically investigated. We hypothesize that participants fail to understand the asymmetry of the response scale of the magnitude estimation method correctly. To follow the logic of the magnitude estimation procedure, participants need to translate their perceptions to ratios. Critically, the scale is not proportional to the intensity of perceptions, and to indicate the same perceived difference in – for example – brightness, smaller differences in judgments are necessary for darker stimuli compared to brighter stimuli. Therefore, we believe that participants assign much more extreme ratings to stimuli lower in intensity compared to the reference stimulus. Accordingly, we hypothesize that the typically observed shape of the psychophysical function and the actual ratings participants use to indicate their perception might not reflect the true association between perceived and objective intensity. Targeting this methodological problem is crucial, as Stevens’ power law represents one of the most prominent approaches to psychophysics. If the typical shape of the power function is biased substantially by characteristics of the method with which it is measured, previous findings need to be interpreted with more caution.

In the following section, we summarize the literature on the estimation of psychophysical functions with regard to Stevens’ power law. In addition, we discuss problems regarding the most common method – magnitude estimation – in more detail and describe how these problems might affect the fitting of power functions. Finally, we present results from three studies in which we examined potential biases in the magnitude estimation procedure. Specifically, in Studies 1 and 2 we investigate the psychophysical functions for brightness perception. In Study 3, we examine whether our criticism generalizes to another dimension (red saturation). In Study 1, we compare a standard magnitude estimation approach to a method in which we reversed the response scale (i.e., higher values indicate darker stimuli). In Studies 2 and 3, we use a unidirectional method to assess the intensity of perceptions. This approach has the advantage that it does not require participants to think in ratios when judging the intensity of the presented stimuli.

### Stevens’ power law

The association between the physical intensity of a stimulus and its perceived intensity has been addressed in various studies beginning in the nineteenth century (see Bauer, 2009, for an overview). While Fechner (1860) postulated that this association is best described by a logarithmic function, Stevens (1957) argued a century later for a power function. In this line of research, one specific method received particular attention: the so-called magnitude estimation method. This approach was developed by S. S. Stevens in the middle of the twentieth century (Stevens, 1957, 1959, 1961). The procedure is based on the comparison of test stimuli to a reference stimulus, to which an arbitrary value is assigned (e.g., 10 or 100). Participants are instructed to rate the perceived intensities of the test stimuli with numbers relative to the reference stimulus.

Using this approach, the relationship between the subjectively perceived intensity and the actual physical intensity of the presented stimuli has been examined for many physical dimensions, including luminance, loudness, red saturation, or vibration. For example, in a magnitude estimation study by Stevens and Stevens (1963), participants had to indicate the perceived intensity of different luminance levels in relation to a reference stimulus (value = 10). Results suggest that the psychophysical function can be described by a power law with exponents ranging between .26 and .33. Other studies investigating luminance as a physical dimension and that are based on the magnitude estimation procedure report similar exponents (Curtis, 1970; Hopkinson, 1960; Marks & Stevens, 1966; Stevens, 1966, 1970; Stevens & Hall, 1966).

### Criticism of the new psychophysics

Because of its simplicity and versatility, the magnitude estimation method – and consequently also the power law – quickly became very popular in psychophysical research. In the following decades, however, the optimism of many researchers declined, as much criticism was voiced about the power law in general and about the application of the magnitude estimation procedure in particular. The most often discussed problem is the fact that many subsequent experiments failed to replicate the power law postulated by Stevens. Several essential problems have been identified so far with regard to the application of magnitude estimations.

First, the assumption that there exists a specific characteristic exponent for each perceptual dimension is questionable. For example, Marks (1974a, 1974b) reports that for loudness perception exponents ranging between .24 and .85 have been found. This example demonstrates that different laboratories using slightly different stimuli and methods sometimes find very different exponents. Exponents of the power function seem to depend substantially on the experimental setup.

Second, some studies found that the power function fits adequately only to averaged data but not to individual data (*averaging effect*). For example, Freides and Phillips (1966) and Steingrimsson and Luce (2006) revealed a lack of fit of the power function when applied to individual data. Generally, differences between psychophysical functions seem to emerge when these are fitted on the individual level rather than on the group level (Bernasconi & Seri, 2016). Other studies, however, also reported a good fit of the power function when fitted to individual as well as to aggregated data (Algom & Marks, 1984, 1990; Marks & Stevens, 1966).

Besides the *averaging effect*, the estimated exponent of the power function strongly depends on the range of stimuli used in the experiments (*range effect*). Engen (1956) was the first to report that larger ranges of stimulus intensity go along with smaller exponents of the power function. Poulton (1968) reviewed the previous literature and revealed that 30% of the variance of exponents can be explained by the range of stimuli applied in the different experiments.

Fourth, the location of the reference stimulus within the stimulus range influences the resulting power functions (*location effect*). The exponent tends to be larger when the reference stimulus is placed in the center of the range and smaller when it is located closer to one of the extremes of the stimulus set. For example, Engen and Levy (1955) reported such a location effect for both brightness and weight judgments (for replications, see Ahlström & Baird, 1989; Fagot & Pokorny, 1989; Pradham & Hoffman, 1963).

Thus, in the past, severe points of criticism concerning the magnitude estimation method have been raised. In the following section, we outline another critical aspect that has not yet been investigated systematically: The requirement of the magnitude estimation method to think in ratios.

### Difficulty of thinking in ratios in the magnitude estimation method

In the magnitude estimation method, an arbitrary reference value is assigned to the intensity of the reference stimulus (e.g., the value 10) and all other stimulus intensities have to be compared to the intensity of the reference stimulus. If, for example in case of brightness perception, a stimulus is perceived to be twice as bright as the reference stimulus, participants have to indicate the number 20 (i.e., 2 × 10), and if the stimulus is half as bright they have to respond with the number 5 (i.e., 10/2). Whereas a multiplication with, or division by, the factor 2 may still be easy to perform, we doubt whether participants are still able to translate their perception to the required factors for more extreme deviations from the reference stimulus. For example, participants have to be aware that a value of 0.1 on the response scale (value of the reference stimulus divided by 100, i.e., 10/100) corresponds to a value of 1,000 (value of the reference stimulus multiplied by 100).

In other words, we assume that a problem arises because of the asymmetry of the response scale, where for stimuli with a lower intensity compared to the reference, the scale ranges from 0 to 10, but for stimuli with a higher intensity, it ranges from 10 to infinity. Thus, for stimuli that are less intensive than the reference only a limited range is available, while for more intensive stimuli an unlimited range is available. Accordingly, the same perceived intensity difference between two stimuli has to be indicated by very small differences in judgments when occurring close to the lower end of the scale, and by very large differences in judgments for higher magnitudes.

To make this point clearer, we will use an example: On a scale anchored by a reference stimulus of value 10 two rather dark stimuli (A and B) might be assigned the numbers 2 and 1. These responses imply that brightness is smaller by a factor of 5 (10/5 = 2) or 10 (10/10 = 1), respectively. Now, two corresponding brighter stimuli (C and D), which are perceived to be 5 or 10 times brighter than the reference, must be assessed with the numbers 50 (10 × 5) and 100 (10 × 10), respectively. Thus, to indicate the same perceived difference in brightness, much smaller differences in judgments are necessary for darker (pairs of) stimuli compared to brighter (pairs of) stimuli. We argue that participants might not be fully aware of this inherent asymmetry of the response scale. Rather, they might remember that their rating for the two dark stimuli A and B differed by only 1, thus thinking in differences rather than in ratios. Accordingly, they may hesitate to respond to C and D with values that are separated by a much larger difference of 50, even though they perceived brightness differences between A and B and between C and D as rather similar.

Thus, we suppose that (at least some) participants, when working on the magnitude estimation task, base their judgments on absolute distances of test stimuli and reference stimuli rather than on their ratios. Following this reasoning, the possible range of ratings for stimuli of lower intensity than the reference value (i.e., values from zero to the reference value) sets an anchor for the maximum values used for very intense stimuli. Accordingly, we expect that participants will typically use the whole scale from 0 to the value of the reference stimulus to assess stimuli that are lower in intensity than the reference stimulus, whereas they might hesitate to use a substantially wider range for stimuli with higher intensity compared to the reference.

In sum, the conceptualization of a response format that requires computing ratios of two perceived intensities might cause problems and bias the validity of the resulting psychophysical functions. Although methods other than the magnitude estimation approach were used in the past and often replicated the power functions (e.g., cross-modality matching: Stevens, 1965; Stevens & Guirao, 1963; forced-choice methods: Ariely, 2001; Chong & Treisman, 2003; magnitude production: Green et al., 1977), the application of such methods is rather scarce. It is further possible that there is a publication bias in favor of those results confirming the typical power functions reported before. Critically, studies comparing results from new approaches to those from magnitude estimation within one experiment are mostly missing. Furthermore, most studies do not compare different psychophysical functions (e.g., power function vs. a linear function), and formal tests of model fit are missing. In our view, testing different methods within one experiment and comparing fits of competing functions is essential to evaluate the adequacy of Steven’s power function for relationships between physical and perceived stimulus intensities.

### The current studies

We conducted a set of three studies, two addressing the physical dimension of luminance (Studies 1 and 2), and one addressing the perception of red saturation (Study 3). In all three studies, a standard magnitude estimation procedure was used in one condition. In this condition, participants assessed the test stimuli’s intensity compared to the reference stimulus with numbers above (below) the reference value of 10 for stimuli of higher (lower) intensity. In each study, we compared this standard method to another method within the same experiment.

In Study 1, we applied a *reversal method*, in which we reversed the direction of the response scale. Thus, participants still rated the perceived brightness of stimuli, but now had to use smaller numbers for brighter stimuli and vice versa. With this first study, we want to demonstrate the general problem of thinking in ratios in the magnitude estimation procedure. We hypothesize that participants will use more (less) extreme ratings for the brightest (darkest) stimuli in the *reversal method* compared to the *standard method* condition because they rather think in absolute distances than in proportions. In Study 2, we compared the *standard method* to a method – which we denote as the *unidirectional method* – that avoids the problem of calculating ratios of perceived intensities. Participants were first asked to indicate whether the target was brighter or darker than the reference stimulus. After this binary choice, participants had to specify how much brighter or darker the target is. In comparison to the standard method, we expected that the unidirectional approach reduces extreme judgments for darker stimuli. As discussed above, we expect that participants in the standard magnitude estimation task should be likely to assign a low value to a very dark stimulus (e.g., assignment of the value 1) while refraining from associating extremely high values (e.g., 100) to very bright stimuli. In the *unidirectional method* condition, on the other hand, participants use the same scale (10 to infinity) for both brighter and darker stimuli. For example, they have to indicate the number 100 independent of whether the stimulus is 10 times as bright or 10 times as dark as the reference stimulus. Accordingly, we expect less curved power functions for the *unidirectional method* (exponents should approach 1) compared to the *standard method* (exponent should be notably below 1)*.*

In Study 3, we again compare the *standard method* to the new *unidirectional method* but use a different physical dimension – red saturation – to test the generalizability of the results obtained in Study 2. We suppose that the function obtained with the new *unidirectional method* will be more linear (or less curved) compared to the function that stems from the typical standard magnitude estimation method.

## Study 1: Standard versus reversal method

In Study 1, we assessed brightness perceptions both with a standard version of the magnitude estimation procedure and with a version with a reversed response scale (i.e., participants had to code darker stimuli with higher and brighter stimuli with lower numbers). We expected that individuals use more (less) extreme brightness judgments for the brightest (darkest) stimuli in the *reversal method* compared to the *standard method* condition.

### Method

#### Participants

A power analysis using G*Power 3 (Faul et al., 2007) was conducted to determine the required sample size. The sample size to detect an effect of large size^2^ (*f* = .40) with a power of .80 and an alpha-error of .05 in a repeated-measures ANOVA setting comprising a within-subject factor with two conditions was 15. We recruited 20 participants from the participants’ pool of a German university with the *hroot* software (Bock et al., 2014). Eighty percent of the participants were students, amongst them 25% studied psychology. All participants completed an informed consent form and were remunerated with course credit or a bar of chocolate. Participants had an average age of 25 years (min = 19, max = 64, *SD* = 9.54) and most of them were female (80%).

#### Stimuli

The luminance stimuli were achromatic (gray) rectangles (width: 960 px, height: 270 px) that were presented on a 17-in. laptop monitor (aspect ratio 16:9) with a screen resolution of 1,920 × 1,080 pixels and a color resolution of 8 bits per channel. We used eight different luminances for test stimuli and one luminance as reference (10 cd/m^2^). Four luminances were brighter and four luminances were darker than the reference luminance. The colorimetric values of the stimuli (see Table 1) were measured by means of a spectroradiometer (Specbos 1201). In each trial of the experiment, the reference stimulus and one test stimulus were presented, centered horizontally in the upper and lower part of the screen, respectively, on a dark background (luminance: 3 cd/m^2^).

**Table 1:** Colorimetric values of the stimuli used in Study 1 and Study 2. Columns x, y, and Y display the CIE xyY values according to the 10° CIE 1964 (Commission Internationale de l'Éclairage, 2006) standard observer, specified relative to a D65 white point

Stimulus	x	y	Y (cd/m^2^)
1	0.303	0.307	1.0
2	0.308	0.314	1.8
3	0.311	0.317	3.2
4	0.314	0.321	5.7
**5**	**0.313**	**0.322**	**10.0**
6	0.315	0.322	17.9
7	0.314	0.321	32.0
8	0.315	0.322	57.2
9	0.313	0.320	100.0

Values in bold indicate the respective values for the reference stimulus

#### Design and procedure

The experiment was administered in a windowless laboratory so that lighting conditions were identical for all participants. The room was dark with the exception of the lighting from a small desk lamp in one corner of the room. Participants were assessed individually. They were seated in front of the laptop at a distance of approximately 60 cm. First, participants had to fill in demographic items and a participant code (to ensure that participants did not take part in more than one of our studies). Meanwhile, participants adapted to the lighting conditions. Then, participants performed both conditions of the task. Task order was counterbalanced across participants.

Instructions for the task were adopted from Teghtsoonian (1965). In our experiment, participants were informed that they had to assess the brightness of the lower rectangle in comparison to the brightness of the upper rectangle, and that the brightness of the upper rectangle remained constant across trials. They were further told that the brightness of the upper rectangle was arbitrarily set to the value 10. In the *standard method* condition, participants read the following instructions:

“If you perceive the lower rectangle as brighter than the upper rectangle, enter a number above 10. If the lower rectangle seems to you, for example, twice as bright as the upper rectangle, enter the number 20 (i.e., 2 × 10). If, on the other hand, the lower rectangle seems half as bright as the upper rectangle enter the number 5 (i.e., 1/2 × 10). There are as many numbers above 10 as there are numbers below 10 because you can also enter decimal places (e.g., 0.5 or 0.125). Do not pay attention to responding as consistently as possible. You do not need to try to remember your responses from the previous trials, but you should assess each rectangle for itself” (translated from German). For the *reversal method* condition, instructions were reversed so that stimuli that were darker than the reference stimulus should be associated with values above 10, and vice versa. Participants completed both conditions, with the order of conditions counterbalanced across participants.

Each trial started with the simultaneous presentation of the reference stimulus and the target stimulus. Participants then had to enter any positive value (possibly including decimal places). There was no time limit for responding. Directly after confirmation of their input, the next pair of stimuli appeared. Each of the eight different luminance levels of the stimuli were presented five times, resulting in a total number of 40 trials per condition. The order of trials was randomized for each participant. On average, the study took 15 min.

### Results

Because participants in the *reversal method* condition were instructed to assign values smaller than the reference to brighter stimuli and vice versa, all responses were recoded using the transformation r′ = 10/r × 10 (e.g., an estimate of r = 20 was recoded to the value of r′ = 5) to allow comparability between the two conditions. Because this transformation is not possible for values that equal zero (which would lead to infinity as the value), we decided to recode all zero values to 0.001. This was also done for zero values in the *standard method* condition, because as the dependent variable the natural logarithm of the ratings was used^3^ and the logarithm of zero does not exist. In sum, 0.68% of the values were recoded.^4^ We used the statistical computing language R for all analyses reported in this article (R Core Team, 2020). Default Bayes Factors with multivariate Cauchy priors on the effects as described in Rouder et al. (2012) are reported alongside the usual ANOVA results. The corresponding Bayes Factors were calculated using the BayesFactor package in R (Morey & Rouder, 2018).

**Fig. 1:**
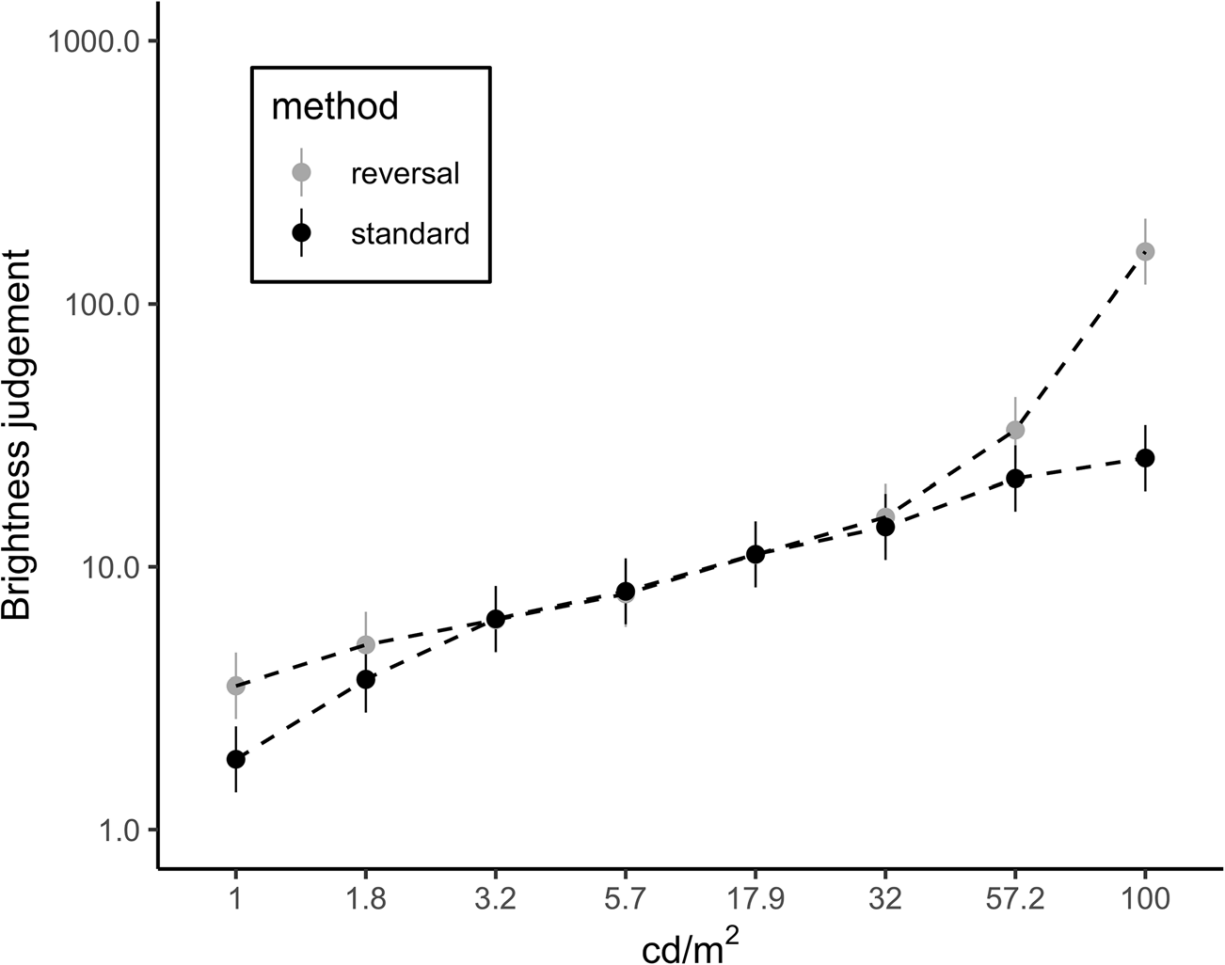
Perceived brightness as a function of luminance level and method (data from Study 1)*.* The y-axis represents a log-scale. Error bars indicate 95% confidence intervals

We ran a within-subjects 2 (method: standard vs. reversal) × 8 (luminance) ANOVA to compare the logarithmized brightness judgments between the two methods. We applied Greenhouse-Geisser correction to account for violations of sphericity in all studies. Besides the main effect of luminance (*F*[1.36, 25.75] = 69.00, *p* < .001, η_g_^2^ = .69, log (*BF*_*10*_) = 685.83), there was a main effect of method (*F*[1, 19] = 50.41, *p* < .001, η_g_^2^ = .09, log(*BF*_*10*_) = 45.67), which was qualified by a significant interaction between method and luminance (*F*[1.47, 28.02] = 13.39, *p* < .001, η_g_^2^ = .17, log(*BF*_*10*_) = 93.18). Figure 1 illustrates the perceived brightness ratings (y-axis) of participants for the eight luminance levels (x-axis) and the two methods (standard vs. reversal). As expected, the brightest (darkest) stimulus was perceived more (less) intensively in the *reversal method* compared to the *standard method* condition.

### Discussion

As hypothesized, the numerical brightness judgments were more (less) extreme for the brightest (darkest) stimuli in the *reversal method* compared to the *standard method* condition. This strongly suggests that the intensity ratings of the participants were affected by the applied method. Participants seemed to fail to understand the asymmetry of the response scale. We suppose that they were thinking in absolute distances rather than in ratios and thus assign more extreme values to stimuli that have to be rated with values smaller than the reference.

## Study 2: Standard versus unidirectional method

Study 1 clearly demonstrated participants’ difficulty to think in proportions regarding the magnitude estimation procedure. In Study 2, we introduced an alternative approach for the measurement of perceived stimulus intensities, which does not require participants to translate the perceptual strengths to ratios. This approach is compared to the standard magnitude estimation method. We further investigated whether the data from the two methods are best fitted by a power or a linear function.

### Method

#### Participants

In Study 2, we again conducted a power analysis using G*Power 3 (Faul et al., 2007) to determine the required sample size. The required sample size to detect an effect of large size^5^ (*f* = .40) with a power of .80 and an alpha-error of .05 in a repeated-measures ANOVA setting with one between-subject factor comprising two groups was 30. Participants (*N* = 34) were recruited from the participants’ pool of a German university with the *hroot* software (Bock et al., 2014). Participants had to complete an informed consent form and were remunerated with course credit or a bar of chocolate. Participants had an average age of 24 years (min = 18, max = 46, *SD* = 5.83) and were for the most part females (85%). Ninety-one percent of the participants were students, with 32% among them having psychology as major.

#### Stimuli, design, and procedure

Stimuli, design, and procedure of Study 2 were for the most part identical to Study 1, with two exceptions: Firstly, we replaced the *reversal method* with a *unidirectional method*. In the *unidirectional method* condition, participants first had to indicate with a binary response whether the presented test stimulus was brighter or darker than the reference stimulus. After this choice, they indicated the strength of their sensation with a numerical judgment. For this condition, we used the following instructions: “If you perceive, for example, the lower rectangle as brighter than the upper rectangle, choose “brighter” as your response to the first question. For the second question, you insert, for example, the number 20 (i.e., 2 × 10) if you perceive the lower rectangle as twice as bright as the upper rectangle (with the value 10). If, on the other hand, you perceive the lower rectangle as being twice as dark as the upper rectangle, you also have to insert 20 (i.e., 2 × 10) as response to the second question, but before you have to choose “darker” as your response to the first question” (translated from German).

Secondly, in contrast to Study 1, we now used a between-subjects design in which each participant was randomly assigned to one of the two conditions (standard vs. unidirectional). Sixteen participants were in the *standard method* condition and 18 participants were in the *unidirectional method* condition. In Study 2, the scales of the two conditions differed essentially: In the standard magnitude estimation task, the scale allowed judgments lower than the reference (10), whereas the *unidirectional method* only allowed inputting numbers larger than 10. As we did not want our participants to get confused between the two conditions, possibly resulting in cross-over effects, we decided to use a between-subjects design*.* The study took about 10 min on average.

### Results

Since participants in the *unidirectional method* condition were only allowed to give ratings larger than or equal to the reference (due to the binary character of the task), it was not possible to directly contrast ratings from both conditions. To make results between the two methods (standard vs. unidirectional) comparable, all responses from trials with darker stimuli (stimulus types 1–4, see Table 1) from the *unidirectional method* were hence recoded using the transformation r′ = 10/r × 10. By doing so, participants’ ratings in the *unidirectional method* condition could be interpreted in the same way as those in the *standard method* condition. We recoded all zero values to 0.001 (0.47% of all trials). Furthermore, we excluded all trials with erroneous responses in the *unidirectional method* condition.^6^ This led to an exclusion of 0.14% of all trials.

**Fig. 2:**
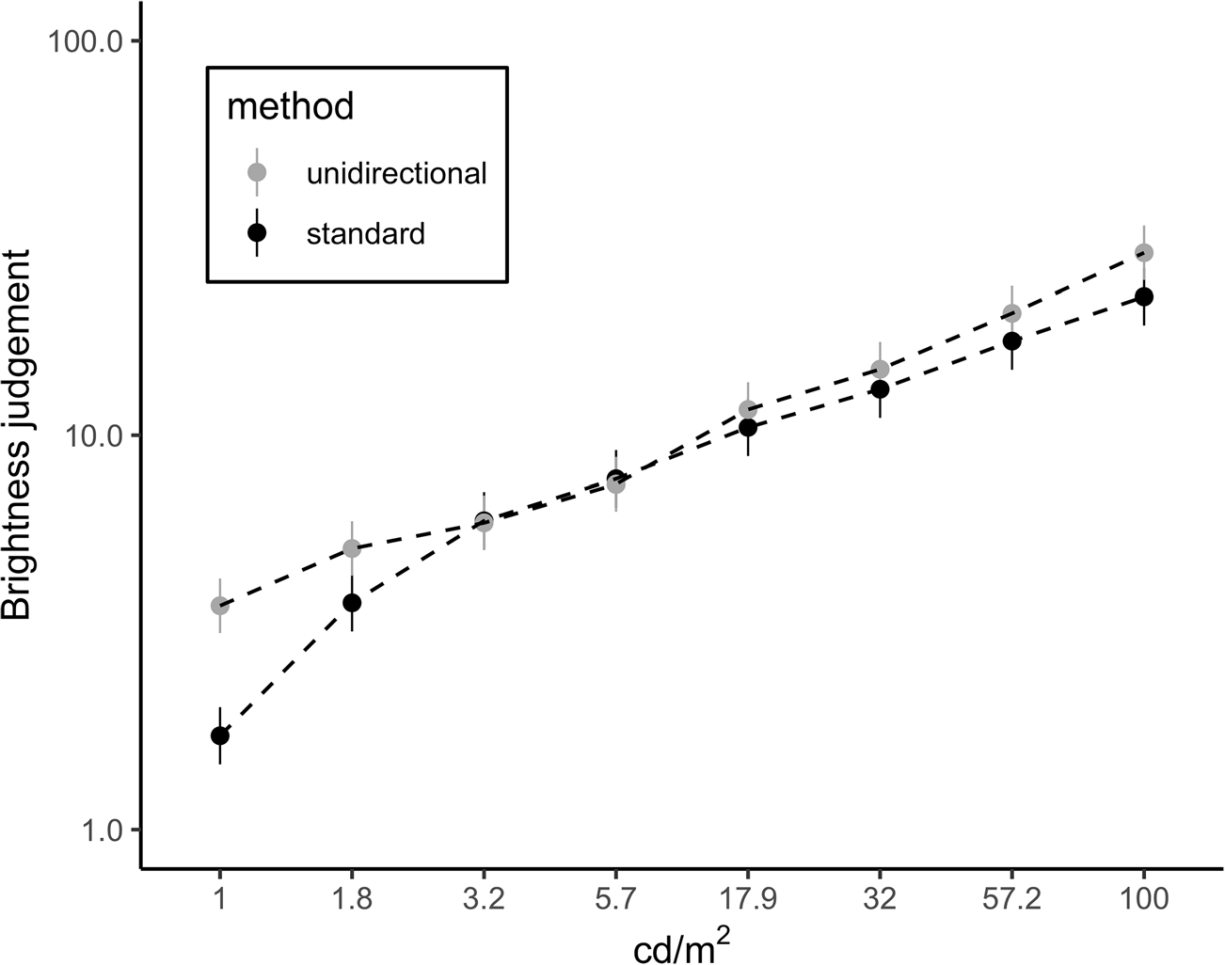
Perceived brightness as a function of luminance level and method (data from Study 2)*.* The y-axis represents a log-scale. Error bars indicate 95% confidence intervals

We entered the logarithmized judgments into a mixed 2 (method: standard vs. unidirectional) × 8 (luminances) ANOVA with method as between-subjects factor and luminance as within-subjects factor. Besides the main effect of luminance, *F*(2.02, 64.70) = 176.75, *p* < .001, η_g_^2^ = .825, log(*BF*_*10*_) = 750.77, there was a main effect of method, *F*(1, 32) = 21.73, *p* < .001, η_g_^2^ = .090, log(*BF*_*10*_) = 3.53, with higher brightness judgments in the *unidirectional* compared to the *standard method* condition. Most interestingly, the interaction between luminance and method was significant, *F*(2.02, 64.70) = 4.71, *p* = .012, η_g_^2^ = .112, log(*BF*_*10*_) = 26.64. Figure 2 illustrates the perceived brightness ratings (y-axis) for the different physical luminance levels (x-axis) and the two methods (standard vs. unidirectional). Whereas there was no essential difference between both conditions for stimuli higher in intensity compared to the reference, judgments for stimuli low in intensity were higher in the *unidirectional method* than in the *standard method* condition. Put differently, relative to the reference value, participants gave less extreme ratings for darker stimuli in the *unidirectional method* compared to the *standard method* condition.

In a second analysis, the psychophysical power functions were estimated directly using a Bayesian mixed-effects model for both methods. We used the following model specification for the hierarchical power law model:

$$ {\displaystyle \begin{array}{c}{y}_j\sim \mathcal{N}\left({b}_{0j}{x}^{b_{1j}},{\sigma}_e^2\right)\\ {}{b}_{0j}={b}_0+{u}_{0j}\\ {}\begin{array}{c}{b}_{1j}={b}_1+{u}_{1j}\\ {}{b}_0\sim \mathcal{N}{\left(0,5\right)}^{\circ}\\ {}\begin{array}{c}{b}_1\sim \mathrm{half}\mathcal{N}{\left(1,0.5\right)}^{\circ}\\ {}{u}_0\sim \mathcal{N}\left(0,{\sigma}_{u_0}^2\right)\\ {}\begin{array}{c}{u}_1\sim \mathcal{N}\left(0,{\sigma}_{u_1}^2\right)\\ {}{\sigma}_{u_0}\sim \mathrm{half}\mathcal{T}\left(3,0,2.5\right)\ast \\ {}\begin{array}{c}{\sigma}_{u_1}\sim \mathrm{half}\mathcal{T}\left(3,0,2.5\right)\ast \\ {}{\sigma}_e\sim \mathrm{half}\mathcal{T}\left(3,0,2.5\right)\ast \end{array}\end{array}\end{array}\end{array}\end{array}} $$

where *y*_*j*_ is the brightness judgment of participant *j* and *x* is the luminance of the test stimulus. Prior distributions marked with an asterisk represent the default priors as defined in the *brms* package in *R* (Bürkner, 2017), i.e., half Student-t distributions with three degrees-of-freedom and a scale of 2.5 for all variance parameters. Prior distributions marked with a circle are the ones we defined.

The models were estimated using the *brms* package (Bürkner, 2017).^7^ For each parameter, we ran four Markov chains with a burn-in phase of 2,000 iterations per chain, and 20,000 post-warmup samples in total for further analyses. The estimated population-level exponent for the *standard method* (*M*_*γ10*_ = 0.44, *SD*_*γ10*_ = 0.05) was smaller than for the *unidirectional method* (*M*_*γ10*_ = 0.55, *SD*_*γ10*_ = 0.07).^8^

Lastly, we directly tested whether the relationship between luminance and judged brightness was best described by a power function or by a linear function. We decided to first fit a Bayesian mixed effects model for both a linear and a power function and to compute the Bayes Factor for the two models afterwards. The Bayes Factor was chosen as the model comparison metric of choice, since it intrinsically penalizes the flexibility of models, even if the models being compared have the same number of parameters (see, e.g., Rouder & Morey, 2012). The linear mixed model has the same number of fixed parameters but one more random effect parameter than the corresponding mixed model for the power law, namely the correlation between random intercept and random slope. We used the following model specification for the hierarchical linear model:

$$ {\displaystyle \begin{array}{c}{y}_j\sim \mathcal{N}\left({\beta}_{0j}+{\beta}_{1j}x,{\sigma}_e^2\right)\\ {}{\beta}_{0j}={\beta}_0+{u}_{0j}\\ {}\begin{array}{c}{\beta}_{1j}={\beta}_1+{u}_{1j}\\ {}{\beta}_0\sim \mathcal{N}\left(0,5\right){}^{\circ}\\ {}\begin{array}{c}{\beta}_1\sim \mathcal{N}\left(0,1\right){}^{\circ}\\ {}\left({u}_0,{u}_1\right)\sim MVN\left(0,\varSigma \right)\\ {}\begin{array}{c}\varSigma =\left(\begin{array}{cc}{\sigma}_{u_0}^2& {\sigma}_{u_0}{\sigma}_{u_1}\rho \\ {}{\sigma}_{u_0}{\sigma}_{u_1}\rho & {\sigma}_{u_1}^2\end{array}\right)\\ {}{\sigma}_{u_0}\sim \mathrm{half}\ \mathcal{T}\left(3,0,2.5\right)\ast \\ {}\begin{array}{c}{\sigma}_{u_1}\sim \mathrm{half}\ \mathcal{T}\left(3,0,2.5\right)\ast \\ {}\rho \sim LKJ(1)\ast \\ {}{\sigma}_e\sim \mathrm{half}\ \mathcal{T}\left(3,0,2.5\right)\ast \end{array}\end{array}\end{array}\end{array}\end{array}} $$

where *y*_*j*_ is the brightness judgment of participant *j* and *x* is the luminance of the test stimulus. Again, default priors are represented by an asterisk, i.e., half Student-t priors and a LKJ-Correlation prior with shape = 1. Prior distributions marked with a circle are the ones we specified. All parameter estimates (fixed effects and the standard deviation of random effects) for both conditions and models, together with the 95% credible intervals, and model fit indices, are displayed in Table 2.^9^

**Table 2:** Mean posterior values (95% credibility intervals in brackets) of all parameters of the power function and linear function (Study 2), separated by condition

	Standard method		Unidirectional method	
Parameter	Power law	Linear model	Power law	Linear model
*γ*_*00*_ / π_00_	0.23 [0.19, 0.28]	0.05 [0.05, 0.06]	0.32 [0.24, 0.40]	0.05 [0.04, 0.06]
*γ*_*10*_ / π_10_	0.44 [0.35, 0.53]	0.20 [0.15, 0.25]	0.55 [0.41, 0.70]	0.28 [0.20, 0.36]
σ(*γ*_*00*_/π_00_)	0.09 [0.02, 0.06]	0.01 [0.01, 0.02]	0.17 [0.12, 0.24]	0.02 [0.01, 0.03]
σ(*γ*_*10*/_π_10_)	0.18 [0.12, 0.27]	0.10 [0.07, 0.14]	0.30 [0.21, 0.45]	0.17 [0.12, 0.25]
σ(ε_ij_)	0.03 [0.03, 0.03]	0.04 [0.03, 0.04]	0.05 [0.05, 0.05]	0.05 [0.05, 0.05]
WAIC	-2456.0 (337.8)	-2358.3 (252.5)	-2138.0 (213.4)	-2151.3 (202.0)
LOOIC	-2509.6 (284.2)	-2379.4 (230.5)	-2148.0 (208.2)	-2155.8 (198.9)
log(Ma.L)	1223.8	1169.2	1053.0	1066.4

*γ*_*00*_/π_00_: fixed effect of proportionality constant/intercept term; *γ*_*10*_/π_10_ fixed effect of exponent/slope term; σ(*γ*_*00*_/π_00_): standard deviation of proportionality constant/intercept term (between participants); σ(*γ*_*10*/_π_10_): standard deviation of exponent/slope term (between participants); σ(ε_ij_): standard deviation of residuals; log(Ma.L): logarithmized marginal likelihood

**Fig. 3:**
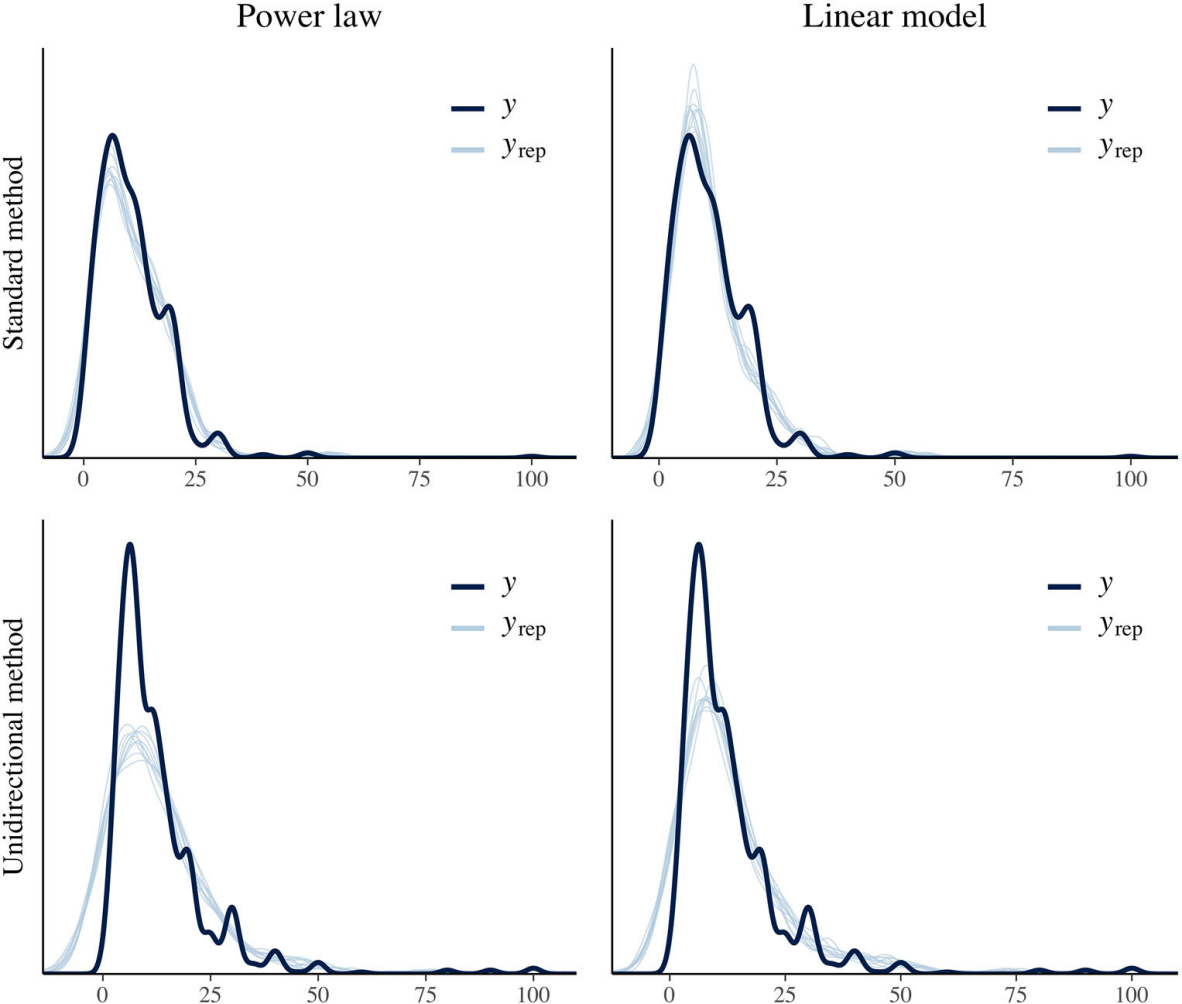
Visualization of the posterior predictive checks for each model (power law vs. linear model) and each condition (*standard method* vs. *unidirectional method*). The dark blue line (y) represents the observed distribution of brightness judgements whereas each of the 10 light blue lines (y_rep_) represents the distribution generated by sampling parameters from the posterior distributions of the respective model

After we fitted both models (linear and power model) for each condition, we computed the Bayes Factor of the two models using the *bridgesampling* package (Gronau & Singmann, 2017). The convergence of the Bayes Factor was ensured by keeping more post-burn-in samples (20,000) than usual and running bridge sampling ten times. In the *standard method* condition, the power law fitted the data much better than the linear model. Logarithmized Bayes Factors showed extreme evidence in favor of the power law (*M*_*log(BF10)*_*=* 54.65, *SD*_*log(BF10)*_ = 0.01). However, for data from the *unidirectional method*, the pattern switched. This time, the logarithmized Bayes Factors revealed extreme evidence in favor of the linear model (*M*_*log(BF10)*_*=* -13.38, *SD*_*log(BF10)*_ = 0.04).^10^ Figure 3 depicts the posterior predictive checks^11^ of the power law and linear model of the *standard method* and *unidirectional method* condition.

#### Additional analyses

To rule out the possibility that the change of the exponent we found when using the *unidirectional method* in comparison to the *standard* magnitude estimation method might be partly due to the preceding binary task,^12^ we recruited an additional sample (*N* = 19, *M*_*age*_ = 21.37, *SD*_*age*_ = 2.37, *range*_*age*_ = 18–27, 79% female, 100% students, 37% psychology as major) that worked on a task that was slightly modified compared to the *unidirectional method*: First, like in Study 2, participants had to execute the binary task. Then, however, they worked on the *standard* magnitude estimation task. In doing so, we were able to directly assess the possible influence of the binary task on the results. If the binary task reduces trial-to-trial effects, the power law fitted to the data of the standard magnitude estimation task without preceding binary task from Study 2 should be different from the power law fitted to the new data. Again, we recoded all values equal to zero to 0.001 in the *binary standard method* condition (0.26% of the trials). When comparing the *standard method* condition with the *binary standard method* condition, we found no difference regarding the estimated exponents in both conditions (the exponents were largely identical; *standard method*: *M*_*γ10*_ = 0.44, *SD*_*γ10*_ = 0.05; *binary standard method*: *M*_*γ10*_ = 0.42, *SD*_*γ10*_ = 0.04). We also analyzed a mixed 2 (method: standard vs. binary standard) × 8 (luminances) ANOVA with method as between-subjects factor and luminance as within-subjects factor. Only the main effect of luminance reached significance, *F*(1.60, 52.89) = 111.11, *p* < .001, η_g_^2^ = .724, log(*BF*_*10*_) = 642.89. There was no significant main effect of method, *F*(1, 33) = .13, *p* = .716, η_g_^2^ < .001, log(*BF*_*10*_) = -1.81, and no significant interaction between luminance and method, *F*(1.60, 52.89) = .24, *p* = .739, η_g_^2^ = .006, log(*BF*_*10*_) = -6.21.

### Discussion

In Study 2, we compared the psychophysical functions from a brightness judgment task between two different assessment procedures. The *standard method* was contrasted with the so-called *unidirectional method*. In this approach, participants first indicated which of the two presented stimuli (reference stimulus or test stimulus) was brighter, and then quantified the difference in perceived brightness. We expected that participants’ judgments would be less extreme for stimuli lower in intensity compared to the reference in the *unidirectional method*.

Results from Study 2 show a strong dependency of the resulting psychophysical functions on the applied method: While the standard magnitude estimation procedure led to a power law with a decreasing slope, data from the *unidirectional method* was best fitted by a linear function. Although the same physical intensities of luminance were used in both conditions, the shape of the curves was clearly different. By transforming judgments from the *unidirectional method* back to the standard scale used by S. S. Stevens, we observed that participants in the *unidirectional method* condition actually gave less extreme judgments for darker stimuli. We argue that participants’ judgments in the *standard method* condition were guided by the perceived implicit boundaries of the scale (0 and 10), making them think in distances rather than in ratios. Thus, participants typically use the whole scale from 0 to 10 to assess stimuli that are lower in intensity than the reference stimulus in the standard magnitude estimation method. However, they hesitate to use a wider range for stimuli higher in intensity compared to the reference because they do not understand the asymmetry of the response scale correctly. The power function in the *standard method* condition was much more curved compared to the function obtained in the *unidirectional method* condition where there is no asymmetry in the response scale. In summary, we argue that the exponent often reported in the literature is, at least to some extent, influenced by the standard magnitude estimation procedure.

The results from Studies 1 and 2 are straightforward. However, the question arises whether the results are generalizable to other physical dimensions. In theory, the problem of thinking in ratios, which is attributable to the magnitude estimation method, should be independent of the physical dimension. Still, we decided to run another study with a different physical dimension. For this purpose, we selected a physical dimension for which an exponent larger than 1 was found when applying the magnitude estimation procedure (in contrast to the exponent of about .33 of the luminance dimension).

## Study 3: Red saturation

To rule out the possibility that the results we obtained in Studies 1 and 2 are specific for luminance, in Study 3 we examined another physical dimension – red saturation. We used the assessment of red saturation for two reasons: First, we were interested in examining a physical dimension for which psychophysical power functions with exponents greater than 1 have been reported. Previous results suggest that the psychophysical function for red saturation can be described by a power function with an exponent of about 1.7 (Panek & Stevens, 1966). Secondly, investigating the subjective perception of different shades of red can be easily implemented on a computer monitor and red saturation can be assessed with the experimental procedures from Study 2. Thus, no differences in the devices or procedures will influence the results. We again expected the function to be much more linear/less curved in the *unidirectional method* condition compared to the *standard method* condition due to the fact that participants should use much more extreme ratings for stimuli lower in red saturation in the *standard method* compared to the *unidirectional method* condition.

### Method

#### Participants

We used the same power analysis as in Study 2. Participants (*N* = 36) were again recruited from a participants’ pool of a German university with the *hroot* software (Bock et al., 2014). Participants gave their informed consent and were compensated with a bar of chocolate. Participants had an average age of 21 years (min = 18, max = 25, *SD* = 1.71) and were mostly female (56%). Ninety-seven percent of the participants stated that they were students, amongst them 36% studied psychology as their major.

#### Stimuli

The stimuli of different red saturation were chromatic rectangles (width: 960 px, height: 270 px) that were presented in the same way as stimuli in the previous studies. We used eight different test shades of red and one reference shade of red (50% saturation). Four shades of red were more saturated and four shades of red were less saturated than the reference shade. The colorimetric values of the stimuli (see Table 3) were measured by means of a spectroradiometer (Specbos 1201) to ensure that the selected shades of red only differed in saturation but not with regard to luminance or hue.

**Table 3:** Colorimetric values of the presented stimuli used in Study 3. Columns X, Y, and Z display the CIE XYZ tristimulus values according to the 10° CIE 1964 standard observer (Commission Internationale de l'Éclairage, 2006), columns L* and h* display the lightness and hue values according to the CIE LCh 1976 system (Commission Internationale de l'Éclairage, 2007), column S displays the saturation values calculated from the LCh 1976 chroma (C*) values: S = C*^2^ / (C*^2^ + L*^2^)^1/2^ · 100% (cf. Lübbe, 2013). L*, S, and h* are specified relative to a D65 white point

Stimulus	*X*	*Y* (cd/m^2^)	*Z*	L*	h* (deg)	*S*
Red 15%	63.00	61.80	58.83	82.81	36.54	14.97
Red 25%	65.86	61.35	53.62	82.56	34.30	24.98
Red 35%	70.32	62.11	48.68	82.97	34.66	35.14
Red 45%	74.07	61.51	43.66	82.65	32.91	44.95
**Red 50%**	**76.56**	**61.72**	**40.56**	**82.76**	**33.72**	**49.79**
Red 55%	79.54	61.89	37.18	82.56	34.21	54.96
Red 65%	86.91	61.82	30.84	82.82	33.33	65.11
Red 75%	97.81	61.90	22.69	82.86	33.41	75.10
Red 85%	117.35	61.79	11.61	82.80	34.34	85.04

Values in bold indicate the respective values for the reference stimulus

#### Design and procedure

The design and procedure of Study 3 were mostly identical to Study 2, except for adjustments in the instructions. Eighteen participants were in the *standard method* condition and 18 participants were in the *unidirectional method* condition*.* For the *standard method* the following instruction was used: “If you perceive the lower rectangle as redder than the upper rectangle, please enter a number above 10. If, on the other hand, you perceive the lower rectangle as less red than the upper rectangle, please enter a number below 10. If the lower rectangle seems to you, for example, twice as red as the upper rectangle, enter the number 20 (i.e., 2 × 10). If, on the other hand, the lower rectangle seems half as red as the upper rectangle, enter the number 5 (i.e., 1/2 × 10). There are as many numbers above 10 as there are numbers below 10 because you can also enter decimal places (e.g., 0.5 or 0.125). Do not pay attention to responding as consistently as possible. You do not need to try to remember your responses from the previous trials, but you should assess each rectangle for itself” (translated from German). For the *unidirectional method* condition we used the following instructions: “If you perceive, for example, the lower rectangle as redder than the upper rectangle, choose ‘redder’ as response to the first question. For the second question, you insert, for example, the number 20 (i.e., 2 × 10) if you perceive the lower rectangle as twice as red as the upper rectangle (with the value 10). If, on the other hand, you perceive the lower rectangle as twice as less red (or half as red) as the upper rectangle, you also have to insert 20 (i.e., 2 × 10) as response to the second question, but before you have to choose ‘less red’ as response to the first question.” (translated from German).

### Results

Like in Study 2, we recoded the responses from trials with stimuli of a lower red saturation compared to the reference (stimulus types Red 15%, Red 25%, Red 35%, and Red 45%, see Table 3) from the *unidirectional* method using the transformation r′ = 10/r × 10. We again recoded all zero values to 0.001 (5.69% of all trials) and excluded all trials with erroneous responses in the *unidirectional method* condition, which led to an exclusion of 2.22% of all trials.

**Fig. 4:**
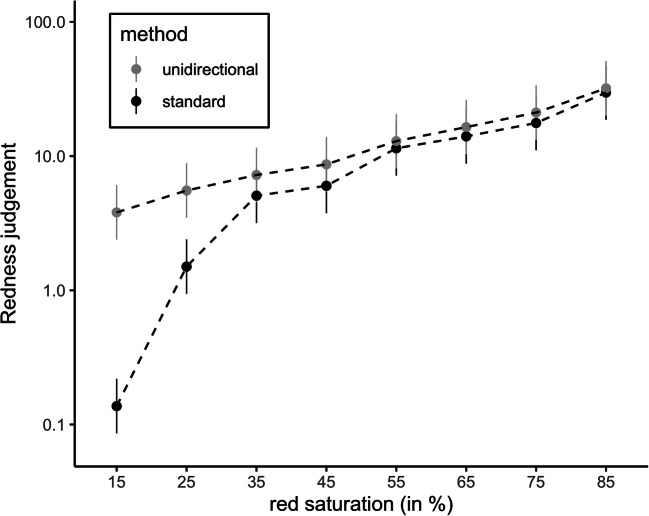
Perceived redness as a function of red saturation level and method (data from Study 3)*.* The y-axis represents a log-scale. Error bars indicate 95% confidence intervals

As in Study 2, we then entered the logarithmized judgments into a mixed 2 (method: standard vs. unidirectional) × 8 (red saturation) ANOVA with method as between-subjects factor and red saturation as within-subjects factor. Besides the main effect of red saturation, *F*(1.60, 54.51) = 56.05, *p* < .001, η_g_^2^ = .567, log(*BF*_*10*_) = 394.70, there was a main effect of method, *F*(1, 34) = 23.31, *p* < .001, η_g_^2^=.123, log(*BF*_*10*_) = 5.39, with higher redness judgments in the *unidirectional* compared to the *standard method* condition. Most interestingly, the interaction between red saturation and method was significant, *F*(1.60, 54.51) = 12.20, *p* < .001, η_g_^2^ = .222, log(*BF*_*10*_) = 117.87. Figure 4 illustrates the perceived redness ratings (y-axis) for the different physical red saturation levels (x-axis) and the two methods (standard vs. unidirectional). Relative to the reference value, participants gave less extreme ratings for stimuli lower in intensity in the *unidirectional method* compared to the *standard method* condition.

As in Study 2, we fitted a Bayesian non-linear mixed effects model for both methods using the same prior distributions and setup as before. The estimated population-level exponent for the *standard method* (*M*_*γ10*_ = 1.99, *SD*_*γ10*_ = 0.23) was higher than for the *unidirectional method* (*M*_*γ10*_ = 1.73, *SD*_*γ10*_ = 0.21).^13^

**Fig. 5:**
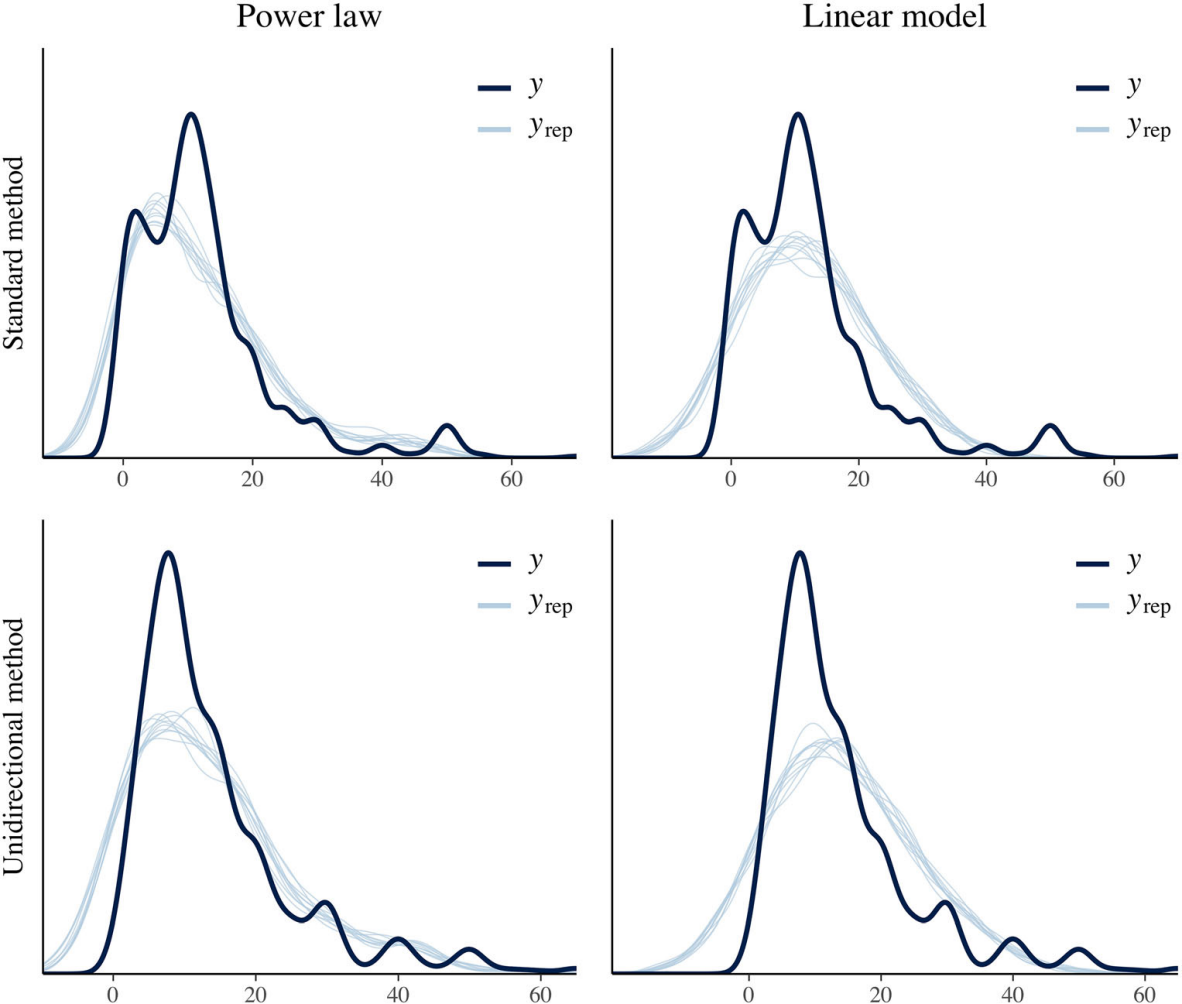
Visualization of the posterior predictive checks for each model (power law vs. linear model) and each condition (*standard method* vs. *unidirectional method*). The dark blue line (y) represents the observed distribution of redness judgements whereas each of the 10 light blue lines (y_rep_) represents the distribution generated by sampling parameters from the posterior distributions of the respective model

We again tested whether the relationship between physical red saturation and perceived redness was best described by a power function or rather by a linear function. All parameter estimates (fixed effects and the standard deviation of random effects) for both methods and models, together with the 95% credible intervals, and model fit indices are summarized in Table 4. For both conditions, the power model fitted the data much better than the linear model. Logarithmized Bayes Factors showed extreme evidence in favor of the power law, with *M*_*log(BF10)*_*=* 199.76, *SD*_*log(BF10)*_ = 0.04, for the *standard method*, and *M*_*log(BF10)*_*=* 204.80, *SD*_*log(BF10)*_ = 0.05, for the *unidirectional method*. Figure 5 depicts the posterior predictive checks of the power law and linear model of the *standard method* and *unidirectional method* condition.

**Table 4:** Mean posterior values (95% credibility intervals in brackets) of all parameters of the power function and linear function (Study 3), separated by condition

	Standard method		Unidirectional method	
Parameter	Power law	Linear model	Power law	Linear model
*γ*_*00*_ / π_00_	0.45 [0.33, 0.56]	-0.07 [-0.09, -0.04]	0.46 [0.34, 0.57]	-0.05 [-0.08, -0.02]
*γ*_*10*_ / π_10_	1.99 [1.52, 2.42]	0.37 [0.30, 0.44]	1.73 [1.31, 2.13]	0.39 [0.30, 0.47]
σ(*γ*_*00*_/π_00_)	0.24 [0.17, 0.35]	0.05 [0.03, 0.08]	0.24 [0.17, 0.35]	0.06 [0.04, 0.09]
σ(*γ*_*10*/_π_10_)	1.01 [0.69, 1.49]	0.14 [0.10, 0.21]	0.91 [0.63, 1.33]	0.18 [0.12, 0.25]
σ(ε_ij_)	0.04 [0.03, 0.04]	0.05 [0.05, 0.05]	0.04 [0.03, 0.04]	0.05 [0.05, 0.05]
WAIC	-2650.2 (85.2)	-2214.4 (85.4)	-2617.9 (73.3)	-2166.5 (71.7)
LOOIC	-2646.7 (86.3)	-2214.0 (85.4)	-2615.8 (73.8)	-2165.9 (71.8)
log(Ma.L)	1269.1	1069.4	1246.3	1041.4

*γ*_*00*_/π_00_: fixed effect of proportionality constant/intercept term; *γ*_*10*_/π_10_ fixed effect of exponent/slope term; σ(*γ*_*00*_/π_00_): standard deviation of proportionality constant/intercept term (between participants); σ(*γ*_*10*/_π_10_): standard deviation of exponent/slope term (between participants); σ(ε_ij_): standard deviation of residuals; log(Ma.L): logarithmized marginal likelihood

### Discussion

In Study 3, we used a different physical dimension – red saturation – to test the generalizability of our findings from the previous studies. Like in Study 2, we found that participants gave more extreme ratings for stimuli lower in intensity in the *standard method* compared to the *unidirectional method*. Although we revealed that the association between objective red saturation and subjective redness perception was best described by a power function in both conditions, the estimated exponent in the *unidirectional method* condition was notably smaller compared to the *standard method* condition. Thus, the power function for the standard magnitude estimation procedure was more curved compared to the one obtained with the *unidirectional method*.

It is important to note that although we found a similar exponent in our new *unidirectional method* compared to the exponent reported in the study by Panek and Stevens (1966), the implemented stimulus material is not entirely comparable between the studies. While a cylindrical color mixer was used in the study by Panek and Stevens (1966), we showed different red saturations on a computer screen. Thus, it is possible that the red saturations were perceived differently in the two studies. Moreover, we fitted the power functions based on individual data. However, and more essentially, the exponent in our *standard method* condition was more extreme compared to the exponent estimated in the *unidirectional method* condition. We argue that the magnitude estimation procedure causes participants to give smaller ratings for stimuli lower in intensity compared to the reference and, therefore, the resulting power functions are curved more extremely compared to the power function we obtained with the *unidirectional method*.

With Study 3, we were able to generalize the findings from the previous two studies that used luminance as physical dimension. Most importantly, we found similar effects for a physical dimension that is typically described by a power function with an exponent smaller than 1 (luminance) and for a physical dimension that is described by an exponent greater than 1 (red saturation). For both physical dimensions, participants gave more extreme ratings for stimuli lower in intensity in the *standard method* condition, thus leading to power functions that are curved more extremely.

## General discussion

With the present research we raise a criticism regarding the adequacy and validity of results from the magnitude estimation approach, which is used very frequently in psychophysics. We argue that individuals are not able to use the scale of the magnitude estimation procedure correctly because of its asymmetry. Whereas for stimuli with a lower intensity compared to the reference, the scale ranges from 0 to 10 (with 10 as the value assigned to the reference stimulus), it ranges from 10 to infinity for stimuli higher in intensity compared to the reference. Participants might not realize that, for example, a value of 1 (value of the reference stimulus divided by 10) corresponds to a value of 100 (value of the reference stimulus multiplied by 10) and that the difference between the values 1 (reference/10) and 2 (reference/5) is much larger than the difference between, for example, the values 15 (reference × 1.5) and 16 (reference × 1.6). Thus, we argue that outcomes of the technique of magnitude estimation might be biased because (some) participants might think in differences rather than in ratios. In our first two experiments, we exemplarily tested this hypothesis for the domain of brightness perception. In the last study, we examined red saturation as a further physical dimension to assess the generalizability of our findings. In all three studies, one condition – which we term the *standard method* condition – was similar to the design of the magnitude estimation method that has often been used in the literature (Stevens, 1957; Teghtsoonian, 1965).

In Study 1, the *standard method* condition was compared to a condition in which the response direction was reversed. Whereas in the *standard method* condition, participants were asked to give ratings lower than the reference (here 10) for darker stimuli and ratings higher than 10 for brighter stimuli, in the *reversal method* condition, lower values had to be entered for brighter stimuli and vice versa. According to the “new psychophysics” (Marks, 1974a, 1974b), in both conditions similar brightness judgments should emerge. However, we found that the brightness judgments for the brightest (darkest) stimuli in the *reversal method* were more (less) extreme compared to the *standard method*. This suggests that the actual experimental procedure influences the judgments of participants.

In Study 2, the *standard method* was compared to a *unidirectional* method. In the *unidirectional method* condition, we eliminated the need to think in ratios by splitting the task into two steps. First, participants indicated whether the presented test stimulus was darker or brighter compared to the reference stimulus. Next, we asked participants to indicate with a number larger than 10 to which degree they felt the stimulus was darker or brighter. A rating of 20, for instance, corresponded to the impression of the test stimulus being twice as dark or twice as bright compared to the reference. As expected, for stimuli darker than the reference, participants in the *unidirectional method* condition gave fewer extreme ratings than participants in the *standard method* condition. More precisely, in the *standard method* condition, participants were more likely to assign extremely low values such as 1, which would correspond to the value of 100 in the *unidirectional method*; however, such high values were rarely used in the *unidirectional method* condition.

In Study 3, we used a different physical dimension – red saturation – to test the stability and generalizability of our findings. We intentionally selected red saturation as the second physical dimension because we wanted to investigate a physical dimension that was found to be best described by a power function with an exponent greater than 1 (in contrast to the exponent smaller than 1 for luminance as physical dimension). We used the same *unidirectional method* as in Study 2 and compared this method to the standard magnitude estimation procedure. Following our predictions, we found evidence for a less extremely curved function between perceived redness and objective red saturation in the *unidirectional method* condition. Moreover, and like in Study 2, participants rated stimuli with lower saturation more extremely in the *standard method* compared to the *unidirectional method* condition. These results are in accordance with our hypothesis that the difficulty in understanding the asymmetry in the magnitude estimation method leads participants to give more extreme ratings for stimuli of lower intensity, which in turn results in a more pronounced curvature of the power functions. Thus, our third study shows that the difficulty to think in ratios not only affects the results from studies that are based on luminance as physical dimension – rather, our hypothesis seems to hold for physical dimensions with exponents that are either smaller than 1 (such as luminance) or greater than 1 (such as red saturation).

Although we did not observe extremely differing exponents for both methods within each study, we want to emphasize that the exponents themselves are not sensitive for the observed differences among the methods. The most pronounced differences in ratings were expected and observed for stimuli lower in intensity compared to the reference. The absolute differences between ratings for stimuli lower in intensity, however, appear small when plotted on a y-axis representing the raw values. Therefore, in order to make the differences in ratings apparent and interpretable, we chose analyses operating on the log-scale. For instance, the mean rating (calculated from the mean log rating) of the stimulus of lowest intensity in Study 3 was .137 in the *standard method* condition, whereas it was 3.808 in the *unidirectional method* condition. On a normal scale representing the raw ratings, the absolute difference between those values would appear small, but when interpreting these values, it means that participants in the *standard method* condition rated the less saturated red stimulus about 30 times lower in intensity compared to participants in the *unidirectional method* condition. Thus, although the estimated exponents might not indicate such a great difference, this does not mean that the two procedures come to similar conclusions.

Birnbaum and colleagues (Birnbaum, 1978; Birnbaum et al., 1989) raised an argument similar to ours many years ago. They compared direct scaling using magnitude estimation to interval scaling to investigate whether this difference in instructions leads to dissimilar sensation functions. Whereas interval scaling predominantly translated into a linear function linking physical to perceived intensity, applying ratio techniques such as the magnitude estimation procedure resulted in power functions. The authors suggested that “when instructed to judge ‘ratios’, the subject cannot make sense of the task and reverts to computing differences” (Birnbaum, 1978, p. 68). In a similar vein, Laming (1984) criticized the fact that there is no fixed rule for assigning ratios of numbers to ratios of stimuli and that the “power law is not intrinsic to the perception of the stimuli, but is contingent on the way in which the subjects are induced to assign numbers to them” (p. 158). Thus, our studies should not be seen as isolated, but rather as providing strong empirical support for an important point of criticism about the magnitude estimation method.

Note that there are also other tasks that despite a different methodological approach resulted in power exponents similar to the magnitude estimation approach. For example, there are tasks in which participants do not get a fixed reference point: Zwlslockl and Goodman (1980; see also Zwlslockl, 1983) asked participants to choose the most appropriate number to represent each stimulus on the participant's own scale. In our eyes, however, this method suffers from the same problems as the usual magnitude estimation task. As no reference point is given, the participants have to choose a reference themselves. Whichever reference value they choose (e.g., 10, 50, 100, or 1,000), they always have to think in ratios since some stimuli need to be assigned lower (higher) values than the (subjective) reference. In our opinion, this assignment of values with respect to a reference is the critical issue.

Another method that has been used in the past is the magnitude production task (DeCarlo & Cross, 1990; Green et al., 1977), in which participants are given a pre-specified list of values to which stimuli have to be associated. Although this method does not predefine a reference point, the selection of values presented to the participants is artificial and there is no clear consensus about the range and numbers of those values that should be used. While picking a reference in the magnitude production task is easy to perform for the participants – since answers are already given via the list of values – it is questionable whether they choose the right reference point. They might choose as reference the point that is closest to the mean value, instead of the median (which would be the correct choice). If this is the case, the magnitude production task may suffer from problems similar to the magnitude estimation task. Interestingly, magnitude production procedures typically produce exponents that are closer to 1 compared to magnitude estimation methods (Laming, 1997). This might also be seen as indirect support for our hypothesis that the difficulty to correctly understand the scale used in the magnitude estimation task influences participants’ judgments about the perceived intensities and affects the shape of the fitted power function.

One further merit of our studies is the stringent testing of different methods against each other. Comparing different methods across studies, as has often been done in the past (e.g., Chong & Treisman, 2003; Stevens & Guirao, 1963), does not allow for clear conclusions because differences might be due to sample characteristics, empirical settings, or other study specifics. Moreover, what is missing from most previous studies is the fact that the power function was not compared to, for example, a linear function. Because of the flexibility of the power function similar power curves might have emerged. A linear function, however, might actually have fitted the data better. In order to quantify the goodness of fit of a function, it is necessary to take fits of competing functions into account.

### Limitations and future directions

The magnitude estimation method that we implemented in our experiments differed slightly from the one used in previous investigations (e.g., Panek & Stevens, 1966; Teghtsoonian, 1965). In previous studies, the reference stimulus was presented first and the following stimuli were shown individually. In contrast, in our experiments, we presented both reference and target stimuli at the same time. We decided for this procedure to ease judgments for the participants. Importantly, when comparing the estimated exponents of the averaged data with those of Stevens’ investigations (e.g., Panek & Stevens, 1966; Stevens & Stevens, 1963), they were virtually identical (.38 vs. .33 for luminance; 1.72 vs. 1.70 for red saturation). Thus, we believe that the slight change in the experimental setup did not influence participants’ behavior substantially.

For the studies presented here, we investigated luminance and red saturation as the domains of interest. There are several reasons for choosing these two physical dimensions. First, luminance was analyzed as the physical dimension of choice by many studies that have been conducted in the past and the associated findings using the magnitude estimation technique are quite consistent. Red saturation was used as the second physical dimension because perceived redness and objective red saturation are described by a power function with an exponent greater than 1. Thereby, we were able to test our hypothesis for different power functions, examining the generalizability of our findings. Lastly, a more pragmatic reason for deciding to use luminance and red saturation is that they can be presented easily in modern labs and both physical dimensions can be assessed with the same procedures. In future studies, one might also examine further physical dimensions (e.g., loudness or vibration). However, we do not expect any differences in the main conclusions from such studies as the general problem (the difficulty to think in ratios) remains the same if the same method (the magnitude estimation method) is used.

A limitation of our studies is that we only examined physical stimuli shown on a computer screen. Although luminance and red saturation were carefully adjusted using a spectroradiometer, it is still not completely clear whether the results obtained in the laboratory experiments translate into real-life light and color sources (e.g., lightbulbs). Furthermore – although we perceive this to be unlikely – findings might be different when another reference is used (e.g., 100). Accordingly, future investigations might check for the stability of psychophysical functions estimated with the *unidirectional method* using different reference values to test the generalizability of our results.

Finally, future research should also investigate more closely whether concerns that have been raised in the past, namely the *range* and the *location effect*, can be addressed adequately by the application of the *unidirectional method*. For example, when using the *unidirectional method*, we assume no *location effect*, i.e., no influence of the choice of the reference point on the final ratings, as participants do not have to think in ratios when using this procedure. Whereas in the magnitude estimation task, the choice of the reference point determines how many stimuli are below or above the reference, no such influence exists for the *unidirectional method*. Using the latter, only participants’ absolute ratings should be shifted up or down with respect to the size of the reference, but the relation of the ratings of each participant should stay the same.

Another interesting future avenue would be to compare models allowing for the notion of “some do and some don’t” as suggested by Haaf and Rouder (2019). The authors argue that it is beneficial if models account for qualitative differences among participants (not all might follow the same underlying mechanism/pattern) and developed ways to analyze such assumptions. In our analyses, the hierarchical power law model already resembles in some way such a mixture model, i.e., it can account for the fact that for some people the relationship between physical and perceived intensity might be linear (exponent = 1) while for others the relationship might rather follow the typical power law (exponent not equal 1). However, we fitted the power law to each condition in Study 2 and Study 3 separately. In a future endeavor, one could go one step further and fit a single model for both conditions. By doing so, the posterior distribution depicting the difference of exponents between the two conditions could even shed more light on the influence of the magnitude estimation procedure on participants’ perception.

### Conclusion

In the present paper, we show that Stevens’ magnitude estimation method entails one major problem: Participants are required to think in ratios for stimuli lower in intensity compared to the reference stimulus. We believe that they rather think in absolute differences because they do not fully understand the asymmetry of the response scale in the magnitude estimation method. This, in turn, might have severe implications for the observed association between actual and perceived intensity of a physical dimension, such as luminance (Studies 1 and 2) and red saturation (Study 3). In Study 1, participants gave more (less) extreme brightness judgments for the brightest (darkest) stimuli in the *reversal method* compared to the *standard method* condition. Furthermore, in both Study 2 and Study 3, we found a more extreme power function for the magnitude estimation method while the exponents were more linear in the *unidirectional method* condition. In the *unidirectional method*, participants were not required to think in ratios. These findings imply that the estimated exponents for the different physical dimensions are dependent on the rating method used. In particular, the standard procedure of magnitude estimation can lead to biased conclusions because participants have difficulties to think in ratios.

#### Open Practices Statement

The data and respective analysis scripts for all experiments as well as a Transparency Report (Aczel et al., 2019) are available at https://github.com/mertensu/thinking-in-ratios. The experiments were not preregistered.

## Footnotes

1 Note that an explicit reference is common, but is not a requisite of the magnitude estimation method. For a discussion of the implications of our findings for magnitude estimation procedures without explicit reference, please refer to the *General discussion* section.

2 We assumed the effect size to be large as we expected the ratings between the two conditions to differ essentially especially for the darkest and brightest stimuli. Note that earlier studies investigating the psychophysical function of luminance based on magnitude estimations (Stevens, 1957; Stevens, 1966; Teghtsoonian, 1965) had sample sizes similar to ours.

3 We chose logarithmized brightness judgments as the dependent variable to account for the actual ratio-scale character of the judgments. Although, for instance, a rating of 4 and a rating of 3 only lead to a nominal difference of 1, such a difference would, for judgments above the reference value of 10, correspond to a difference of 10 × (10/3 - 10/4) = 8.33.

4 We reanalyzed the data excluding all trials with values equal to zero to rule out the possibility that the recoding of the zero values to 0.001 biased the results in some way. Importantly, we obtained similar results for the respective analyses in all studies.

5 We again assumed a large effect size as we expected the ratings between the two conditions to differ profoundly, especially for stimuli that were lower in intensity compared to the reference.

6 We decided to delete these values because it is not possible to unequivocally decide whether these errors represent the real perception of participants or accidental wrong key presses. However, given the small percentage of errors, this had no influence on our findings.

7 To achieve a more efficient and faster sampling, we aimed to scale both predictor and outcome variables to have roughly unit-scale (Stan Development Team, 2020). In order to not change the underlying relationship though, we reduced all variables by a factor of 100. By doing so, sampling was more robust and chains converged much faster while at the same time it did not influence the estimated exponents/slopes in any way. The same was done in Study 3.

8 We additionally estimated the exponents for both conditions using the aggregated data (median data). When averaging the data, the estimated exponent for the *standard method* (*M*_*γ10*_ = 0.38, *SD*_*γ10*_ = 0.04) was again smaller than in the *unidirectional method* (*M*_*γ10*_ = 0.52, *SD*_*γ10*_ = 0.09).

9 We computed both the widely applicable information criterion (WAIC) and the leave-one-out cross-validation information criterion (LOOIC) as model fit indices (Vehtari et al., 2017). Both methods measure the out-of-sample accuracy of predicted values with smaller values depicting better model fit. We additionally computed the logarithmized marginal likelihoods (model evidences) that represent the (logarithmized) probability of the data given the respective model (Etz, 2018). Here, higher values indicate a better model fit.

10 Although the estimated exponent in the *unidirectional method* (.55) does not directly indicate a linear function, we found evidence for the latter. We believe it is most likely that the linear function benefits from not being restricted to pass the origin (0,0). We could confirm this by fitting the power function with an additive constant. In this case, the logarithmized Bayes Factors showed evidence in favor of the power law (*M*_*log(BF10)*_*=* 6.87, *SD*_*log(BF10)*_ = 0.02). However, the estimated exponent was .82 [0.64, 1.01], also indicating a more linear relation.

11 In posterior predictive checks, replicated data are simulated under the fitted model and these data are compared to the observed data. Thus, discrepancies between simulated and observed data can be identified (Gelman, 2014).

12 The reason for implementing the *unidirectional method* was to eliminate the influence of thinking in ratios on the brightness judgments. However, as the binary task is preceding the actual estimation task, the initial response of the participants might not be captured (DeCarlo, 1992) and using this method might reduce the magnitude of a trial-to-trial sequence effect (Cross, 1973; Ward & Wolff, 1973). Stewart et al. (2005) emphasized with their relative judgment model (RJM) that participants use information from previous stimuli for their judgment of current stimuli. As a consequence, participants’ judgments are biased towards previous judgments due to assimilation processes. More precisely, the RJM puts forward the argument that only the immediately preceding trial is used for judging the current stimulus and no other information from the longer history of judgments is used. In sum, following the logic of the RJM model, it is highly plausible that memory effects influence the judgments in the magnitude estimation procedure. Such effects might be smaller for our two-step procedure.

13 When using the averaged data, the estimated exponent for the *standard method* (*M*_*γ10*_ = 1.72, *SD*_*γ10*_ = 0.28) was also higher than in the *unidirectional method* (*M*_*γ10*_ = 1.56, *SD*_*γ10*_ = 0.26).
